# An Intelligent Multi-Sensor Variable Spray System with Chaotic Optimization and Adaptive Fuzzy Control

**DOI:** 10.3390/s20102954

**Published:** 2020-05-22

**Authors:** Lepeng Song, Jinpen Huang, Xianwen Liang, Simon X. Yang, Wenjin Hu, Dedong Tang

**Affiliations:** 1 School of Electrical Engineering, Chongqing University of Science and Technology, Chongqing 401331, China; slphq@163.com (L.S.); jinpenhuang@163.com (J.H.); gollum0@163.com (X.L.); hwjok@126.com (W.H.); cqtangdd@163.com (D.T.); 2 School of Engineering, University of Guelph, Guelph, ON N1G 5H1, Canada

**Keywords:** multi-sensor measurement, data analysis, adaptive fuzzy control, chaos optimization, variable spray, double closed-loop control

## Abstract

During the variable spray process, the micro-flow control is often held back by such problems as low initial sensitivity, large inertia, large hysteresis, nonlinearity as well as the inevitable difficulties in controlling the size of the variable spray droplets. In this paper, a novel intelligent double closed-loop control with chaotic optimization and adaptive fuzzy logic is developed for a multi-sensor based variable spray system, where a Bang-Bang relay controller is used to speed up the system operation, and adaptive fuzzy nonlinear PID is employed to improve the accuracy and stability of the system. With the chaotic optimization of controller parameters, the system is globally optimized in the whole solution space. By applying the proposed double closed-loop control, the variable pressure control system includes the pressure system as the inner closed-loop and the spray volume system as the outer closed-loop. Thus, the maximum amount of spray droplets deposited on the plant surface may be achieved with the minimum medicine usage for plants. Multiple sensors (for example: three pressure sensors and two flow rate sensors) are employed to measure the system states. Simulation results show that the chaotic optimized controller has a rise time of 0.9 s, along with an adjustment time of 1.5 s and a maximum overshoot of 2.67% (in comparison using PID, the rise time is 2.2 s, the adjustment time is 5 s, and the maximum overshoot is 6.0%). The optimized controller parameters are programmed into the hardware to control the established variable spray system. The experimental results show that the optimal spray pressure of the spray system is approximately 0.3 MPa, and the flow rate is approximately 0.08 m^3^/h. The effective droplet rate is 89.4%, in comparison to 81.3% using the conventional PID control. The proposed chaotically optimized composite controller significantly improved the dynamic performance of the control system, and satisfactory control results are achieved.

## 1. Introduction

Each year, millions of tons of pesticides are used in agriculture around the world, which has not only resulted in serious pollution of almost all freshwater resources, but also has posed a threaten to populations, environments, and ecosystems [1,2]. China is no exception and is troubled by pesticide abuse that has caused a number of serious environmental and ecological problems. The direct dose of pesticides to targeted pest accounts for only 0.03% of the pesticides used [3]. Compared to the conventional application techniques that may be a great waste of pesticides, variable spray technology is there able to help raise the utility of pesticides to a much higher level, making it possible to improve the future crop yield and quality in a positive way. Variable spray is one of the research hotspots aiming at pesticide application in precision agriculture in industry. Kim and Son [4] divided the number of light cooperative robots in line with the size of the farmland work space, so as to realize variable farmland spraying operations. Gupta et al. [5] applied intelligent agricultural equipment for path planning, automatic walking, sowing and variable spray. Luo et al. [6] realized variable spraying of concrete by checking the thickness and area of sprayed concrete, thereby to improve the variable spraying effect of concrete. Ruiz-Rodriguez et al. [7] studied the influence of the spray angle of the nozzle on the global and local spray characteristics with the help of the fuel injection and fluid dynamics inside the cylinder in attempt to improve the spray oil combustion effect. This is followed by Giménez et al. [8] who managed to use electronic spray to control the droplet diameter aiming at the study of biological effects. Berenstein and Edan [9] investigated how the nozzle diameter and nozzle angle position of the fixed-point spraying device may affect the variable nozzles to highly reduce the amount of pesticides applied. Lottes et al. [10] used a full convolutional network of encoders and decoders to identify image information and implemented the spray weeding through the image information. Law and Cooper [11] sprayed preservatives or water through the induction charging nozzle of the high-efficiency electrostatic spray device to prolong the preservation of fruit and vegetable freshness. Blanco et al. [12] introduced the directed air tower and multi-headed fan tower to improve the spray effect of orchards, to improve the utilization rate of pesticides. Hołownicki et al. [13] installed spray systems on both sides of the spray head. The variable air assist system worked to continuously adjust the air volume in real time to get better spray effect. Markle et al. [14] employed the ‘gear up/throttle down’ methodology to greatly reduce the drift of mist droplets in the air. The above-mentioned research efforts are in connection with the workspace, intelligent equipment, spray characteristics, electronic spray, inductive charging nozzles, directed air tower and multi-headed fan tower, in order to improve the spray effect.

Many studies [15,16,17,18,19] have used the laser particle size sensor and the displacement sensor to study the angle of the spray nozzle, the nozzle and crop spacing, and they even made some changes on the structure of the nozzle to improve the effect of the spray operation. Martin [20] developed the first commercial variable-hole air nozzle. The flow rate of the nozzle varied in a range of approximately 10 times. In this way, the variable spray could be achieved. Subsequently, especially with pulse width modulation (PWM), Liu et al. [21] designed a microprocessor and a flow automatic control system with PWM to control the start of solenoid valve. The flow of each pipeline was thus controlled to achieve variable flow control of each nozzle. Gu et al. [22] obtained the variable flow rate of the solenoid valve by controlling the PWM duty cycle. This kind of control is impressive in changing the flow rate quickly and accurately, but the constant pressure is a catch to achieve changes in spray angle and droplet size during operation. Faiçal et al. [23] and Zhu et al. [24] later counted on the SLR camera and the laser particle size sensor to track the UAV flight path and laser guided sprayer to get better variable sprays. Needham et al. [25] explored a high-frequency (10 KHz) PWM signal to regulate the current in the valve solenoid and further to control the fluid pressure. A low-frequency (10 Hz) PWM signal was used to control the nozzle flow as per the duty cycle to achieve flow control. In addition, Fritz et al. [26], Qiu et al. [27], and Sun et al. [28] have investigated the nozzle type, hole size, spray pressure, spray rate, spray direction, and liquid conductivity to increase the amount of droplet deposition with the combination of pressure sensors and speed sensors. Finally, many studies [29,30,31,32,33,34] have worked on the laser particle size instrument sensor to get the most deposition from online mixed drugs, air spray, electrostatic spray, and even the changed droplet size. This is especially true when it comes to electrostatic spray. By developing a VR spraying device based on ultrasonic sensors, Bowen et al. [35] illustrated theoretically and experimentally that the charged droplet group would generate an electric field in space to facilitate the deposition of pesticide droplets. Law et al. [36] from the University of Georgia applied inductive charging to carry out electrostatic spraying of pesticides in 1966, and concluded that the combination of electrostatic spraying and air-blasting sprays may help droplet deposition and penetration. The best fog droplet size for electrostatic spray was also recommended with other conclusions. Yule et al. [37] investigated the relationship between charging voltage and spray angle. The results demonstrated that the spray angle was positively related to the charging voltage. When the charging voltage reached the breakdown voltage of kerosene, the spray angle was reduced sharply. Results obtained from the above studies have provided solutions to some typical problems, such as the shape of the nozzle, the distance between the nozzle and the crop, the spray angle, and how to get the best spray effect and the largest deposition amount from different sizes of droplets. Nonetheless, these researchers somehow didn’t emphasize the aspect of system control, i.e., under what control conditions of the whole system the spray droplets are most suitable and beneficial to the deposition of droplets. To start with, the micro-flow variable spray system is a large-lag, time-varying, multi-perturbation nonlinear system. The expected requirements for droplet size and dose of the system are difficult to meet. Then, dose and droplet size may vary from one crop and area to another. The flow rate, system pressure, and other parameters need to be optimal for the droplets sprayed under the conditions, which are most favorable to the deposition of droplets. Subsequently, it must be ensured that the system does not overshoot and the response time is short. That is, the corresponding system without overshooting does not emit excessive dose, whilst in the short response time, the system does not spray insufficient dose accordingly. These are the key issues to solve the variable spray from the perspective of the control system.

At present, four control methods are available to be widely applied in agricultural variable spray and related fields: PID control [38,39,40,41,42,43], fuzzy control [44,45,46,47,48,49,50,51], neural network control, and the corresponding intelligent control [52,53,54,55,56,57,58]. It is a known fact that the PID parameters, once adjusted, can be hard to re-correct. The membership function applied in the fuzzy control is basically in the form of triangle and normal distribution. Upon selection of these two membership functions, the parameters corresponding to the fuzzy control are almost fixed beyond correction. As to the variable spray control system, each stage has its different characteristics and requirements. At the initial stage, the system requires the fastest response time. When it is closing to the required dose (expected value) of the crop, the system needs to be very careful in overshooting. Here the stability of the system plays a major role in controlling the overshoot. Even after the system enters the stable stage, the control requirements of the system flow, the droplet diameter, and speed will be even more different in the presence of external interference. Precisely, the system is so complex and changeable that the control strategy and method must be changed in time to achieve the purpose of real-time control. In light of this, it is proposed to achieve a fast response by using a Bang-Bang relay controller [59] at the initial stage of the variable spray control system. The Bang-Bang control function is designed with time optimization (that is, the system meets the predetermined requirements with a period of shortest time consumed) [60]. After acquiring the required dose of the crop, the system stability and accuracy become the priority indicators. At this time, the controller works by means of adaptive fuzzy nonlinear PID control [61]. Finally, the stable system needs to maintain control of the flow rate, the droplet diameter, and speed at the same time. The double closed-loop mode (i.e., pressure closed-loop and flow closed-loop) is adopted as an alternative control method to make sure that the flow rate of variable spray is adjusted accurately and maximum control of droplet deposition is achieved [62,63]. In this work, external interference may be more effectively suppressed by leveraging Li’s experience of applying adaptive fuzzy control to the four-stage inverted pendulum, whereas the membership function of adaptive fuzzy control was designed with variable parameter PID [61]. Based on the above arguments, this paper mainly attempts to use the Bang-Bang control and adaptive fuzzy PID control theory to further improve the accuracy of control effect of variable spray. The simulation results show that, compared with the PID control strategy, the variable domain adaptive fuzzy PID control strategy enhanced the control accuracy of the system by 4 to 5 times [64]. Additionally, the control stability was greatly improved, and the system had effectively no overshoot. Combining the roles of the pressure sensor, flow sensor, and Doppler particle dynamics sensor, the experimental variable spray particles were measured to an optimum value of 100–300 microns [65,66,67].

This paper is organized as follows: Section 2 describes the construction of a multi-sensor variable spray system. The core of the system consists of two parts. Part 1 is the structure of the control loop, and part 2 is the structure of the controller that consists of Bang-Bang and adaptive fuzzy nonlinear PID. The controller consists of flow loop and pressure loop. Section 3 is to design adaptive fuzzy nonlinear PID controller and chaos optimization algorithm. Section 4 simulates and compares the conventional PID controller and the composite control system. In Section 5, a variable spray control system is built up to conduct experiments to verify the control effects of the control system. Finally, Section 5 provides the conclusions of the study.

## 2. The Developed Variable Spray System

**Figure 1:**
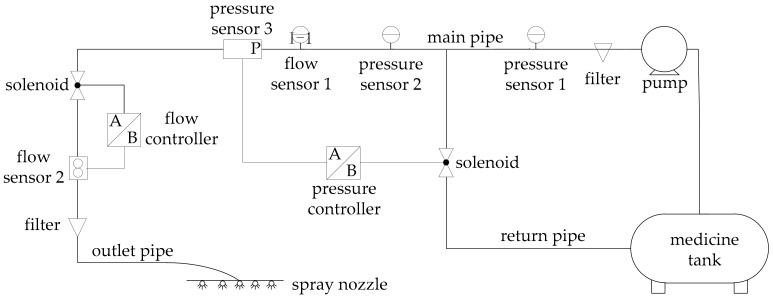
The spraying pipeline system with double closed-loop control (flow controller and pressure controller).

Currently, the variable spray system is controlled in 3 ways, i.e., open loop, single loop, and multi-loop. In comparison, the variable spray system has more parameters and interference factors, and they interfere with each other and are strongly coupled with each other, making it difficult for open-loop control and single-loop control to achieve the desired control effect. Therefore, this system is designed as a double closed-loop control system. The specific structure is shown in Figure 1.

When the spraying operation is performed, the tractor is equipped with a power output shaft that drives the drug pump through a V-belt pulley. The medicine pump is designed with a water inlet and a water outlet. The liquid is drawn from the box under the action of the pump, and it is divided into two pathways. One is the bypass return pipe, which is used to release the pressure in the pipe, and the other passes through the flow controller and is sprayed by the nozzle to water the crops. A filter is installed on the main road to ensure that the pipeline is unobstructed. In addition, a pressure sensor is fitted to detect the pressure and taken as an input to the pressure controller. A pressure control valve is installed in the bypass return pipe to receive and execute the output signal of the pressure controller to achieve the purpose of controlling pressure. The outlet pipe is equipped with a flow sensor and a flow control valve to ensure a constant flow at the outlet. Change in flow rate will affect system pressure, and flow rate will also be affected when system pressure changes. Flow and pressure are mutually coupled and affect each other, so double closed loops are needed to achieve precise control.

**Figure 2:**
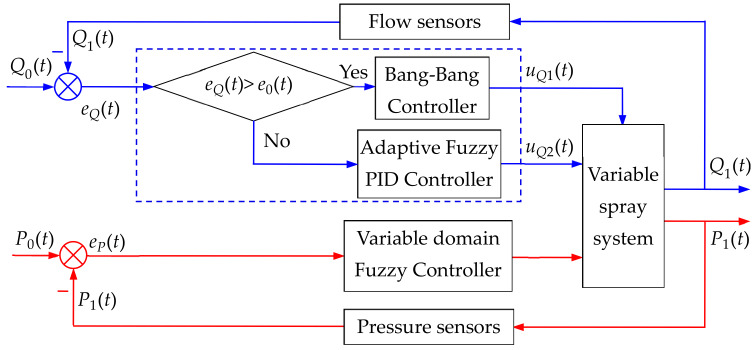
Diagram of variable spray in the control structure on double closed-loop.

The variable spray system is expected to have accurate and rapid discharge of the pesticide amount required by the crop, and the sprayed pesticide droplets are deposited on the surface of the plant to the utmost extent. Meeting both requirements at the same time can be quite difficult by solely controlling the spray amount or the pipe pressure. Using only the single-loop control system would be difficult to achieve accurate control of both flow and pressure. It remains difficult to reach this goal due to the mutual constraint between the two quantities. To handle the above setbacks, a double closed-loop control system was therefore designed to control the flow rate and pressure separately (as shown in Figure 2). The outer closed-loop is for flow control (blue loop), and the inner closed-loop is for pressure control (red loop). The outer loop feeds the flow value through the flow sensor, and the inner loop feedback is the pressure value obtained from the pressure sensor. In this way, the exact amount of spray and the maximum amount of deposition on the plant surface are achieved at the same time.

The goal of the inner closed-loop for pressure control is to keep the pressure of the main circuit as the required level is at the maximum amount of droplet deposition. Different crops pressure may vary when the maximum amount of droplets is deposited, yet on the same crop the pressure is the same, and this pressure value is defined as $P_{0}$ [68]. Therefore, the inner closed-loop for pressure control was designed as a constant value (as shown in Figure 2). That is, the desired pressure from the spray pipe was compared with the actual pressure of the pipe measured by the pressure sensor, and the pressure deviation and its rate of change were obtained. Then, these two parameters were used as input quantities of the controller to control the backflow control valve after the variable domain fuzzy controller operation, thus the pressure of the main spray line eventually became the required pressure when the droplet deposition is the largest. Since the pressure loop was a fixed value for the control over the same crop, a good effect may be achieved by controlling the loop with the variable domain fuzzy.

Note: $Q_{0}\left(t\right)$ is the spray quantity required by the crop,$Q_{1}\left(t\right)$ is the measured spray quantity of the flow sensor, $e_{Q}{(t)=Q}_{0}{(t)\;-\;Q}_{1}\left(t\right)$ is the difference between the spray quantity required by the crop and the measured spray quantity of the flow sensor, which is referred to as the deviation of the spray quantity; ${\dot{e}}_{Q}\left(t\right)=de_{Q}\left(t\right)/dt$ (${\dot{e}}_{Q}\left(t\right)$ is called the rate of change of spray quantity deviation; $P_{0}\left(t\right)$ is the pressure quantity required by the main pipe, $P_{1}\left(t\right)$ is the measured pressure quantity of the pressure sensor,$e_{p}{(t)=P}_{0}{(t)\;-\;P}_{1}\left(t\right)$ is the difference between the pressure quantity required by the main pipe and the measured pressure quantity of the pressure sensor; $e_{0}\left(t\right)$ is the given value of the spray error. Figure 2 is the core technology of the variable spray control, aiming to achieve the on-demand variable spray. The main principles are as follows:

- During the operation, the variable spray device acquires the current position information through the AgGPS 132, and compares the AgGPS 132 position signal with the spray prescription map pre-stored in the computer to obtain the prescription amount at the position. The prescription amount, along with the speed of the implement measured by the speed sensor and the width of the implement, are used as three input quantities. Then, the spray amount required for the crop ($Q_{0}\left(t\right)$) is obtained through a fitting function (a fitting function by the quadratic regression orthogonal experiment). Subsequently, the difference between the spray amount and the actual spray amount of the crop measured by the flow sensor is used to obtain the deviation, $e_{Q}\left(t\right)$*,* of the spray amount and the change rate ${\dot{e}}_{Q}\left(t\right)$ thereof.
- Taking the deviation $e_{Q}\left(t\right)$ of the spray quantity and the change rate ${\dot{e}}_{Q}\left(t\right)$ of the deviation as the input values of the controller, the output values are used to control the flow valve, so that the flow valve will spray the pesticide quickly and accurately according to the actual needs of the crop. Here, double closed-loop control is adopted to get accurate control of spray volume and maximum control of droplet deposition.

The goal of the closed-loop flow control is to maintain the amount of spray at any time, making it available to accurately control the variables according to the needs of the crop, that is, the real-time control. The closed-loop flow control is viewed as the core of the controller design. The key technical problem of the variable spray control system is to design the controller with the best indicators in the shortest possible time. Conventionally, the variable spray in the agricultural control system is dimmed for its large hysteresis, nonlinearity, and time-varying characteristics. It is necessary to fundamentally solve the contradiction between dynamic quality and stability accuracy. Just using PID control and fuzzy control will not lead to better control effect. To tackle this issue, this work was performed with the combination of the Bang-Bang relay controller and adaptive fuzzy PID control methodology (as shown in Figure 2).

The flow sensor measured the actual injection amount at a large error (i.e., |$e_{Q}\left(t\right)$| ≥${\;e}_{0}\left(t\right)$, $e_{0}\left(t\right)$ is the critical value between large error and small error, and empirically taken as 85% of the steady state value = $K_{d}$, $e_{0}\left(t\right)$ is chaos optimization results). The control switch selected the output controlled by the Bang-Bang controller as the control quantity $u_{Q1}\left(t\right)$. At a small error |$e_{Q}\left(t\right)$| ≤ $e_{0}\left(t\right)$, the output of the adaptive fuzzy nonlinear PID controller was selected as the control amount $u_{Q2}\left(t\right)$. Here, the PID controller has the proportional gain $K_{p}$, integration gain $K_{i}$, and derivative gain $K_{d}$ that were modified online by the adaptive fuzzy controller (i.e., the fuzzy gain self-tuning controller). The parameters of the PID controller were automatically adjusted online to further enhance the nonlinear PID controller performance. Thus, flexibility to adapt to changes in the parameters of the control system and changes in operating conditions are satisfied.

## 3. The Multi-Sensor Fuzzy Control System

According to the description of the multi-sensor variable spray system, three input signals of the crop prescription map, the driving speed, and the working width of the agricultural implement were sampled to the controller through the sensor. The controller was programmed to output the control voltage, which is responsible for controlling the opening of the electric regulating valve. The opening degree in turn determined the flow rate of the pesticide sprayed by the variable system. There would be a long delay from the three input signals to the output signal. In addition, the amount of the pesticide changed in response to the demand information of different crops. Apparently, the multi-sensor control object had the characteristics of large inertia, nonlinearity, strong lag, and multi-time variation, and it was difficult to obtain an accurate mathematical model. The conventional control methods are based on expert experience and multiple trial and error methods, so it can be truly hard to control the multi-sensor variable spray system. Now the chaos optimization fuzzy control algorithm provides a better solution to this problem.

### 3.1. Design of Adaptive Fuzzy PID Controller for Flow Closed Loop System

#### 3.1.1. Design of Fuzzy Controller

Using a fuzzy controller with two-dimensional input and one-dimensional output, the error is $e_{Q}(t)=Q_{0}(t)\;-\;Q_{1}\left(t\right)$, where $Q_{0}\left(t\right)$ is the ideal pesticide amount, and $Q_{1}\left(t\right)$ is the actual amount of pesticide needed for crops. The fuzzy processing transformed the error $e_{Q}\left(t\right)$ and the error change rate ${\dot{e}}_{Q}\left(t\right)$ of the system into the corresponding fuzzy domain, getting them quantized. The quantization factor of the error $e_{Q}\left(t\right)$ mapped to the fuzzy domain is $k_{e}$, and for the error change rate ${\dot{e}}_{Q}\left(t\right)$, the quantization factor is $k_{\dot{e}}$.

#### 3.1.2. Adaptive Fuzzy Nonlinear PID Controller: Structure Design

**Figure 3:**
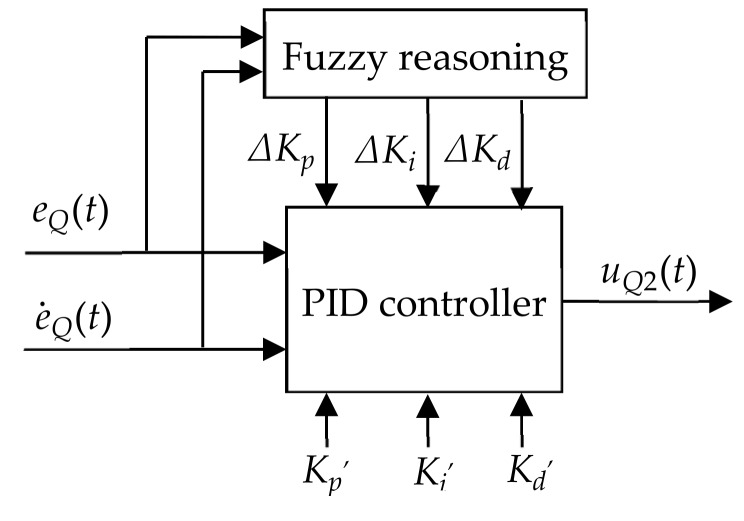
Diagram of the adaptive fuzzy nonlinear PID controller. Note: $K_{p}{}^{\prime }$ is the initial value of proportional gain; $K_{i}{}^{\prime }$ is the integral initial value; $K_{d}{}^{\prime }$ is the differential initial value.

An adaptive fuzzy nonlinear PID controller was composed of a fuzzy system controller and a PID controller, as shown in Figure 3.

The PID parameters were designed using the PID algorithm. By calculating the current system deviation $e_{Q}\left(t\right)$ and the deviation change rate ${\dot{e}}_{Q}\left(t\right)$, the adaptive fuzzy method was used to find the increments Δ$K_{p}$, Δ$K_{i}$ and Δ$K_{d}$ of the PID parameters. $K_{p}{}^{\prime }$, $K_{i}{}^{\prime }$, $K_{d}{}^{\prime }$ were obtained through chaos optimization, so the parameters of the PID controller $K_{p}$, $K_{i}$, $K_{d}$ are:

(1) $$\begin{array}{l} K_{p}=K_{p}^{\prime }+\Delta K_{p}\\ K_{i}=K_{i}^{\prime }+\Delta K_{i}\\ K_{d}=K_{d}^{\prime }+\Delta K_{d} \end{array}$$

#### 3.1.3. Fuzzy Membership Function

**Figure 4:**
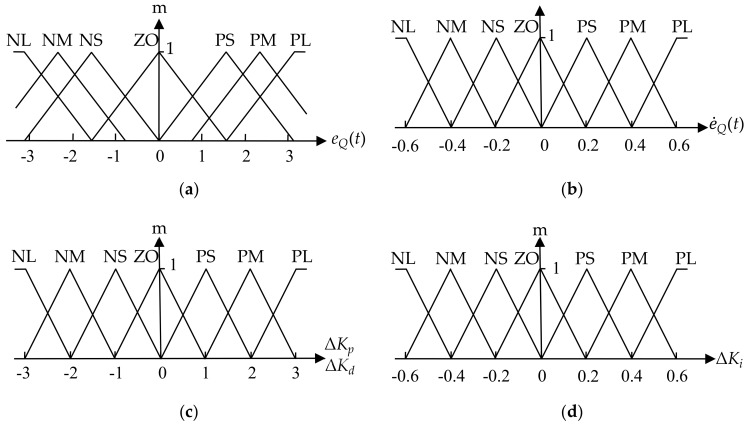
Membership functions for the input and output variables (**a**) $e_{Q}\left(t\right)$ of the target flow; (**b**) ${\dot{e}}_{Q}\left(t\right)$ of the target flow; (**c**) the output Δ$K_{p}$ and Δ$K_{d}$; (**d**) the output Δ$K_{i}$.

According to the actual control requirements, the error $e_{Q}\left(t\right)$ of the target flow and the actual flow of the fuzzy controller had the value of the linguistic variable taken as negative large (NL), negative medium (NM), negative small (NS), negative zero (NZ), positive zero (NZ), positive small (PS), positive medium (PM), and positive large (PL). With respect to the error rate ${\dot{e}}_{Q}\left(t\right)$, the proportional increment Δ$K_{p}$, the integral increment Δ$K_{i}$, and the differential increment Δ$K_{d}$, the value of the linguistic variable was taken as negative large (NL), negative medium (NM), negative small (NS), zero (ZO), positive small (PS), positive medium (PM), positive large (PL). The error $e_{Q}\left(t\right)$, Δ$K_{p}$ and Δ$K_{d}$ quantization levels were taken as {−3, −2, −1, 0, +1, +2, +3}. The change rate of the error ${\dot{e}}_{Q}\left(t\right)$ and Δ$K_{i}$ quantization levels are all taken from {−0.6, −0.4, −0.2, 0, +0.2, +0.4, +0.6} For each variable, the membership functions are shown in Figure 4. Due to the presence of liquid turbulence, the flow rate was relatively unstable, and each detected flow value may fluctuate. To ensure the basic stability of the flow rate, $e_{Q}\left(t\right)$ was allowed to have a small deviation within the allowable range of the spray concentration error, the membership function of $e_{Q}\left(t\right)$ was set to be thin in the middle section and dense at both ends. The adjustment of the error rate of change also takes into account the stability and rapidity of the system response. The membership function of the error rate of change ${\dot{e}}_{Q}\left(t\right)$ was therefore set to a uniformly distributed triangle function. When the deviation was large, fast response was needed. For a small deviation, the response accuracy needed to be increased and the overshoot reduced. Therefore, the membership function of Δ$K_{p}$, Δ$K_{d}$ and Δ$K_{i}$ were therefore set to a uniformly distributed triangle function.

#### 3.1.4. Fuzzy Rules and Decision-Making Methods

The adaptive fuzzy nonlinear PID controller works on the basis of the PID algorithm. Following the fuzzy control rules, the *e* and ${\dot{e}}_{Q}\left(t\right)$ were used for fuzzy reasoning, and the fuzzy matrix table was queried for parameter adjustment [59].

- When $e_{Q}\left(t\right)$ was large, the system had good fast tracking performance by taking a larger $K_{p}$ and a smaller $K_{d}$. Meanwhile, to avoid a large overshoot, the integral action should be limited, usually taken as *K_i_* = 0;
- When $e_{Q}\left(t\right)$ and ${\dot{e}}_{Q}\left(t\right)$ were in the medium level, the system had a smaller overshoot by taking a smaller $K_{p}$, where the value of $K_{d}$ had a greater influence on the system, and an adapted $K_{i}$ should be taken;
- When $e_{Q}\left(t\right)$ was small, the system had better steady-state performance by taking the larger $K_{p}$ and $K_{i}$ values. The value of $K_{d}$ should be appropriate to avoid oscillation near the equilibrium point.

A suitable fuzzy rule table was established for the proposed controller based on the accumulated expertise in long-term practice [59]. The fuzzy control rule tables of the three parameters, Δ$K_{p}$, Δ$K_{i}$ and Δ$K_{d}$, were naturally obtained and given in Table 1, Table 2 and Table 3, respectively.

The fuzzy controller worked with the centroid method (COG) to defuzzify the fuzzy subset. In the process of defuzzification, the weighted average was taken to obtain the exact value of the three PID tuning parameters that were output after defuzzification. The PID controller output an accurate value signal to the motor according to Δ$K_{p}$, Δ$K_{i}$ and Δ$K_{d}$ of the real-time setting output, and the actual flow rate of the flow valve was controlled by the motor armature voltage.

### 3.2. Design of Variable Domain Fuzzy Controller of Pressure Closed Loop System

Using a fuzzy controller with two-dimensional input and one-dimensional output, the error variable of pressure $e_{p}{(t)=P}_{0}\left(t\right)-P_{1}\left(t\right)$, where $P_{0}\left(t\right)$ is the ideal system pressure, and $P_{1}\left(t\right)$ is the actual amount of pressure needed for system. ${\dot{e}}_{p}\left(t\right)$ is the error change rate of system pressure. The error, error rate of change and output UF are defined as (negative large, negative small, zero, positive small, positive large) i.e., (−2, −1, 0, 1, 2) five grades by the unequal distance method. The fuzzy processing transformed the $e_{p}\left(t\right)$ and ${\dot{e}}_{p}\left(t\right)$ of the system pressure are:

(2) $$\left|E\right|=\left\{\begin{array}{cc} 0 & \left|e_{P}\leq n_{1}\right|\\ 1 & \left|n_{1}\leq e_{P}(t)\leq n_{2}\right|\\ 2 & \left|n_{2}\leq e_{P}(t)\right| \end{array}\right.$$

(3) $$\left|EC\right|=\left\{\begin{array}{cc} 0 & \left|{\dot{e}}_{p}(t)\leq m_{1}\right|\\ 1 & \left|m_{1}\leq {\dot{e}}_{p}(t)\leq m_{2}\right|\\ 2 & \left|m_{2}\leq {\dot{e}}_{p}(t)\right| \end{array}\right.$$

where $e_{p}\left(t\right)$ is the same symbol as *E*; ${\dot{e}}_{p}\left(t\right)$ is the same symbol as *EC*; 0 < *n*_1_, *n*_2_, *m*_1_, *m*_2_ < 1.

Because under different pressures, the required working state of the system is different, and the weighted value of error and error change rate should be different. When the system error is large, the main task of the controller is to quickly eliminate the error. Therefore, the larger α value, that is, to strengthen the error weight, should be taken to accelerate the rapid response of the system. On the contrary, when the system error is small, the main task of the controller is to make the system stable as soon as possible. Therefore, a smaller value should be taken, that is, to strengthen the weight of the error change rate, in order to improve the stability of the system. In order to meet the different requirements of the system in different states, three correction factors are introduced. Its analytical expression is as follows:

(4) $$u_{f}=\left\{\begin{array}{ll} <\alpha_{1}E+(1-\alpha_{1})EC> & E=0\\ <\alpha_{2}E+(1-\alpha_{2})EC> & E=\pm 1\\ <\alpha_{3}E+(1-\alpha_{3})EC> & E=\pm 2 \end{array}\right.$$

where < > is the rounding operation, *α*_1_, *α*_2_, *α*_3_ are the correction factors, and the value range is between 0 and 1.

The function of defuzzification is to transform the fuzzy control quantity UF into the precise control quantity $u_{p}\left(t\right)$, and introduce the proportion factor $k_{f}$. The defuzzification transformation is:

(5) $$u_{P}(t)=k_{f}u_{f}$$

where $k_{f}$ is the defuzzification factor.

### 3.3. Chaos Optimization Algorithm for Controller

**Figure 5:**
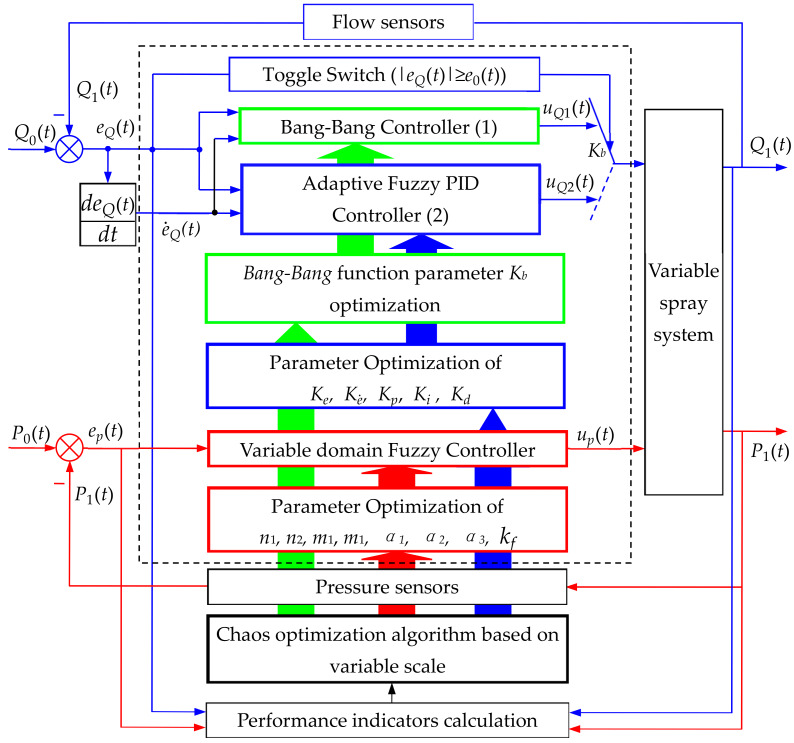
Parameter optimization structure of controller (1), controller (2) and controller (3). Note: *K_b_* is the critical value of $u_{Q1}(t)\;$ q switched to $u_{Q2}\left(t\right)$. $K_{e}$ is the quantization factor of $e_{Q}\left(t\right)$ and $K_{\dot{e}}$ is the quantization factor of ${\dot{e}}_{Q}\left(t\right)$; *K* is the transfer switch.

The control core of the variable spray, as shown in Figure 2, is adaptive fuzzy PID controller and variable domain fuzzy controller. The parameter optimization structure diagram of the controller is shown in Figure 5. The performance of the controller was determined by the parameters of the controller itself. The parameters of the conventional Bang-Bang control and adaptive fuzzy nonlinear PID control are determined by expert experience. The experience may be quite different from each other owing to the subjective factors of experts themselves. Therefore, it was difficult to ensure that the control system would have better control performance. Chaos is a relatively common phenomenon in nonlinear systems. Chaotic motion is highlighted in ergodicity and randomness. It can traverse all states in a certain range without repeating according to its own laws. This means that we can use chaotic variables as a scientific and feasible way to optimize the parameters of adaptive fuzzy controllers. To this end, this work introduced chaotic variables into the adaptive fuzzy control process for parameter optimization. Firstly, based on the performance indicators of the system, the chaotic variables were used to roughly search for the parameters of the adaptive fuzzy controller, and followed by the refined chaos search. The finally searched parameters of adaptive fuzzy controller were viewed as the global optimal parameter values [68,69,70]. The chaotic parameter optimization flow chart is shown in Figure 5.

Use logistic map to generate chaotic variables for optimized search by:

(6) $$x_{n+1}=\mu x_{n}(1+x_{n}),\;x_{n}\in [0,1]$$

where $x_{n}$ is a chaotic series, and *μ* is a control parameter. When *μ* = 4, the system is in a chaotic state. The chaos optimization system was set to two cycles, a rough cycle and a fine cycle, where the rough cycle was responsible for finding a suboptimal solution and a fine cycle was performed near the suboptimal solution to find an optimal solution of the spray system.

Step 1: Construct an evaluation function. The objective function was designed and optimized according to the ITAE objective function criterion composite with the dynamic and static performance of the comprehensive evaluation control system. When the system overshoot and rise time and error are fully considered, the optimization objective function is designed as:

(7) $$J=w_{1}\int_{0}^{t}\frac{t\left|e(t)\right|}{\max(e(t))}dt+w_{2}\sigma +w_{3}t_{r}$$

where *J* is the objective function; $w_{1},\;w_{2},\;w_{3}$ are positive weighting factor, $w_{1}+w_{2}+w_{3}=1$; *σ* is the overshoot of the spray system; and $t_{r}$ is the rise time of the spray system.

Step 2: initialization parameters and initial value selection. Let the iteration flag of the chaotic variable be *K*, *N*_1_ as the time(s) of coarse search (first search), *N*_2_ is the time(s) of fine search (Secondary Search), the controller parameter vector is $L=(K_{e},\;K_{\dot{e}},K_{p},K_{i},K_{d},K_{b},n_{1},n_{2},m_{1},m_{2},\alpha_{1},\alpha_{2},\alpha_{3},k_{f})$ and the chaos variable corresponding to each parameter is $x_{i}=\left(x_{1},\ldots ,x_{14}\right)$ the optimal value of the current chaotic variable is $x_{i}^{*}=\left(x_{1}^{*},\ldots ,\;x_{14}^{*}\right)$ the optimal value of the parameter variable of the current design controller is $L^{*}=(K_{e}^{*},K_{\dot{e}}^{*},K_{p}^{*},K_{i}^{*},K_{d}^{*},K_{b}^{*},n_{1}^{*},n_{2}^{*},m_{1}^{*},m_{2}^{*},\alpha_{1}^{*},\alpha_{2}^{*},\alpha_{3}^{*},k_{f}^{*})$, and the current optimal objective function value $J^{*}$ is initialized to a larger number. 6 different values in the (0, 1) interval are the initial values of the chaotic variable $x_{i}=\left(x_{1},\ldots ,\;x_{14}\right)$. Yet the selection of each initial value must satisfy the relationship $x_{i}\;\neq \;1-\;x_{j}$.

Step 3: Map the chaos variable domain to the controller parameter domain. Chaos variable $x_{i}\;$= ($x_{1}$,…,$x_{14}$) has the domain (0,1), compared to the domain of the controller parameter[$a_{i}$,$b_{i}$], where $a_{i}$ is the lower limit of the value range of the parameter to be optimized, $b_{i}$ is the upper limit of the value range of the parameter to be optimized. Map chaotic variable $x_{i}$ through linear transformation to controller parameter domain $L_{i}$ by following a mapping relationship, i.e.,

(8) $$L_{i}=a_{i}+(b_{i}-a_{i})x_{i}\;\;(i=1,\cdots ,14)$$

where *L*_1_ to *L*_14_ correspond to parameters $K_{e},\;K_{\dot{e}},\;K_{p},\;K_{i},\;K_{d},\;K_{b},\;n_{1},\;n_{2},\;m_{1},\;m_{2},\;\alpha_{1},\;\alpha_{2},\;\alpha_{3},\;k_{f},$ respectively.

Step 4: Find the suboptimal solution in the first cycle. Let the number of rough searches be *N*_1_, and the number of loops is *K*. Each parameter has a chaos trajectory that is taken as initial value of each parameter to be optimized $x_{i}$. In a limited number of searching optimizations, the performance indicators of this set of values that meet the performance indicators are compared to achieve the best performance indicators, that is, that number of groups with *J* as the minimum value is taken as the suboptimal value of each parameter adjustment factor J*. This marks the completion of the rough search for chaos optimization. That is

while *k* < *N*_1_, calculate the performance index *J*

if ${J\;<\;J}^{*}$, then $x_{i}^{*}{=x}_{i}$*,* ${J=J}^{*}$,$L_{i}^{*}{=L}_{i}$

k=K+1, $x_{i}=4x_{i}\left(1+x_{i}\right)$, end

where $x_{i}^{*}$ is the suboptimal value of the chaotic variable, and $L_{i}^{*}$ is the suboptimal value of the parameters of the current design controller, $J^{*}$ is the current suboptimal objective function value.

Step 5: Narrow down the value range of each variable. In the variable-scale chaos optimization algorithm, there is a need for the search space of the optimization variables to be continuously reduced according to the search process. The variable space reduction coefficient is a parameter representing the degree of reduction of the search space of the optimization variable in each “Secondary Search” process. This work used t to represent it. Where controller parameter domain [$a_{i}$,$b_{i}$] kept narrowed into a domain [$a_{i}^{r+1}$,$b_{i}^{r+1}$], and the specific solution process of this domain is:

(9) $$\left\{\begin{array}{l} a_{i}^{r+1}=x_{i}^{r}-t(b_{i}^{r}-a_{i}^{r})\\ b_{i}^{r+1}=x_{i}^{r}+t(b_{i}^{r}-a_{i}^{r}) \end{array}\right.$$

where *i* indicates the *i*-th variable, *r* is the number of times of “Secondary Search”,$x_{i}^{r}$ represents the optimal value, and $b_{i}^{r}$,$a_{i}^{r}$ indicates the upper and lower limits of the of the *i*-th variable obtained from the *r*-th “Secondary Search”, respectively, $b_{i}^{r+1}$ and $a_{i}^{r+1}$ indicates the upper and lower limits of the *i*-th variable in the *r*+1-th “Secondary Search”.

The range of *t* in Equation (7) is (0, 0.5), and when the search space is large, *t* should take a larger value to ensure the speed of the search. When the search space is small, *t* should take a smaller value to ensure the accuracy of the search. Therefore, *t* in Equation (7) is designed as:

(10) $$t=\frac{1}{2}\exp(-\frac{1}{\left|b_{i}^{r}-a_{i}^{r}\right|})$$

Step 6: Select the adjustment coefficient for “Secondary Search”. Such adjustment coefficient refers to the fine-tuning value based on the suboptimal point obtained by rough search, and the new chaotic variable is:

(11) $$x_{i,k}^{r+1}=(1-\alpha )x_{i}^{r}+\alpha x_{i,k}^{r}$$

The new chaotic variable is used for “Secondary Search”. In this paper, the adjustment coefficient for “Secondary Search” is expressed by *α*. Among them, *k* represents the *k*-th chaotic search;$\;x_{i,k}^{r+1}$ represents the chaotic variable used by the *i*-th variable in the *k*-th chaotic search in the *r*+1-th “Secondary Search”; $x_{i}^{r}$ represents the suboptimal solution of the *i*-th variable obtained by the *r*-th search; $x_{i,k}^{r}$ represents the chaotic variable used by the *i*-th variable in the *k*-th chaotic search in the *r*-th “Secondary Search”.

It can be seen from Equation (7) that *α* should be a number related to *r*, the number of times of “Secondary Search”, and the value of *α* should be a smaller number to ensure that fine-tuning in the vicinity of the suboptimal point. At the same time, as the number of “Secondary Search” increases, the results of optimization will continue to approach the true value, and *α* should be continuously reduced to ensure the accuracy of the results of optimization.

For this purpose, the determination formula of parameter *α* is proposed as:

(12) α=exp(−r)

Step 7: Find the optimal solution in the second cycle. Set *N*_2_ as the number of times of the second cycle of variable-scale chaos search, and take $x_{i,k}^{r+1}$ into the Logistic mapping, and linearly map it to the value interval of the design variables [$a_{i}^{r+1}$,$b_{i}^{r+1}$], where $a_{i}^{r+1}$ is the lower limit of the value range of parameter to be optimized in “Secondary Search”, $b_{i}^{r+1}$ is the upper limit of the value range of the parameter to be optimized in “Secondary Search”. In the “Secondary Search”, map the chaotic variable $x_{i,k}^{r+1}$ through linear transformation to the controller parameter domain. The corresponding variable is $L_{i,k}^{r+1}$, and $L_{i,k}^{r+1}$ indicates the controller variable used in the *k*-th chaotic search of the *i*-th variable in the *r*+1-th “secondary search”. The variables in the chaos domain are mapped to the variables in the controller parameter domain and transformed to:

(13) $$L_{i,k}^{r+1}=a_{i}^{r+1}+(b_{i}^{r+1}-a_{i}^{r+1})x_{i,k}^{r+1}$$

while *k* < *N*_2_ calculate the performance indicator *J*

if J < J*, then $x_{i,k}^{r+1*}=x_{i,k}^{r+1}$, J* = J, $L_{i,k}^{r+1*}=L_{i,k}^{r+1}$, end

*k* = *k* + 1, $x_{i,k}^{r+1}=4x_{i,k}^{r+1}(1\;-\;x_{i,k}^{r+1})$, end

where $x_{i,k}^{r+1*}$ is the optimal value of the final chaotic variable, and $L_{i,k}^{r+1*}$ is the optimal value of the final controller parameter, J* is the final optimal objective function value.

**Figure 6:**
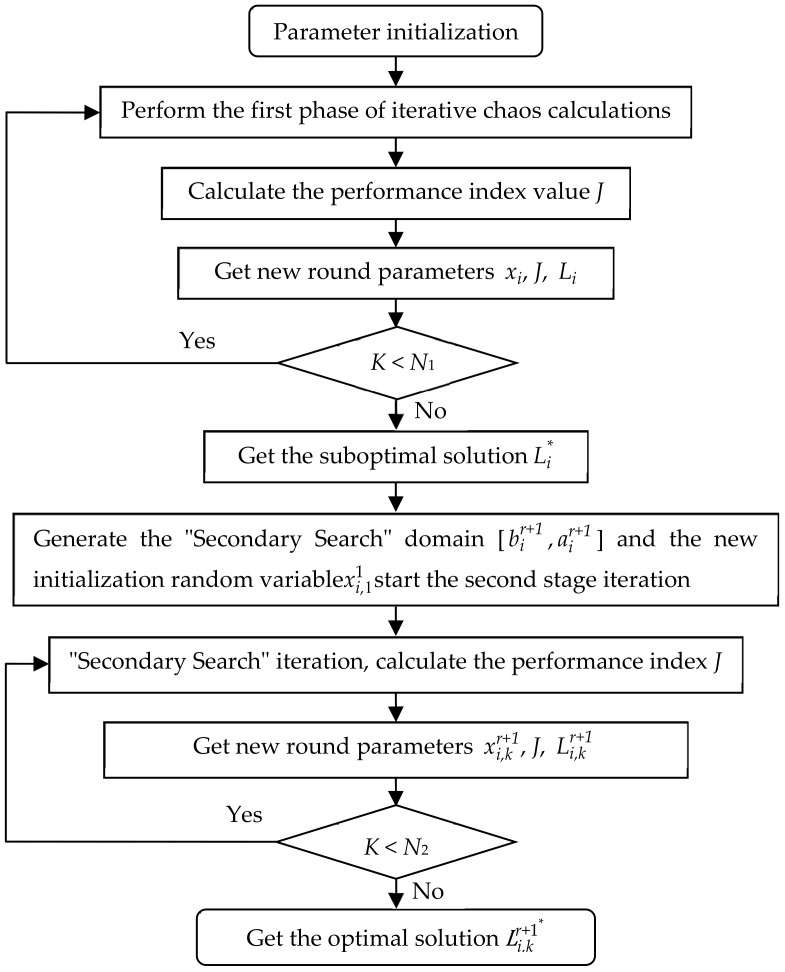
Workflow of chaos optimization.

Step 8: Find the optimal solution for the spray system. Repeat Step 5 and optimization ends after several times, and the optimal design variable is L*, the optimal objective function value is J*, and the variable spray adaptive fuzzy controller parameters have reached the optimal. The flow chart of chaos optimization algorithm based on variable scale is shown in Figure 6.

### 3.4. Simulation for the Control System

**Figure 7:**
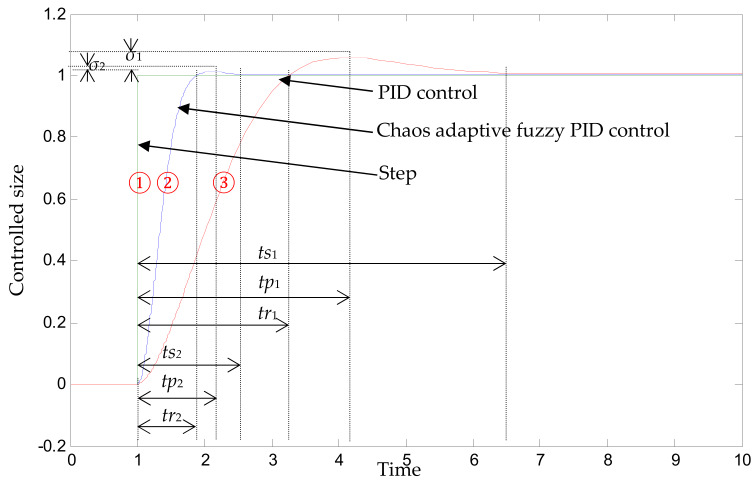
Simulation curve.

Under the action of unit step signal, the variable spray control system model was built in Simulink. In the variable spray control system, the Bang-Bang controller parameters were combined with adaptive fuzzy PID controller parameters to study the basic characteristics of dynamic response, anti-interference and robust, which were then compared with the conventional PID control scheme, as shown in the simulation curve of Figure 7: ① is the waveform of the given signal, ② is the output waveform of the Bang-Bang control and self-optimization using chaos adaptive fuzzy PID control, ③ is the PID control output waveform. From this simulation waveform, the following conclusions can be drawn:

First, both the fuzzy PID and the conventional PID were able to reach the set value under the step response; second, the rise time of conventional PID control was app. *tr*_1_ = 2.2 s, the peak time was app. *tp*_1_ = 3.1 s, the adjustment time was app. *ts*_1_ = 5 s. After chaotic optimization, the rise time of composite controller was app. *tr*_2_ = 0.9 s, the peak time was app. *tp*_2_ = 1.1 s, the adjustment time was app. *ts*_2_ = 1.5 s, which indicated that the chaotic optimized composite controller was faster than the conventional PID adjustment; Third, the maximum overshoot of conventional PID adjustment was *σ*_1_% = 6.00%, and the maximum overshoot of the chaotic optimized composite controller was app. *σ*_2_% = 2.67%. This indicates that the composite controller has smaller punching and better stability.

## 4. Experimental Results of the Variable Spray System

### 4.1. Experimental Setup

**Figure 8:**
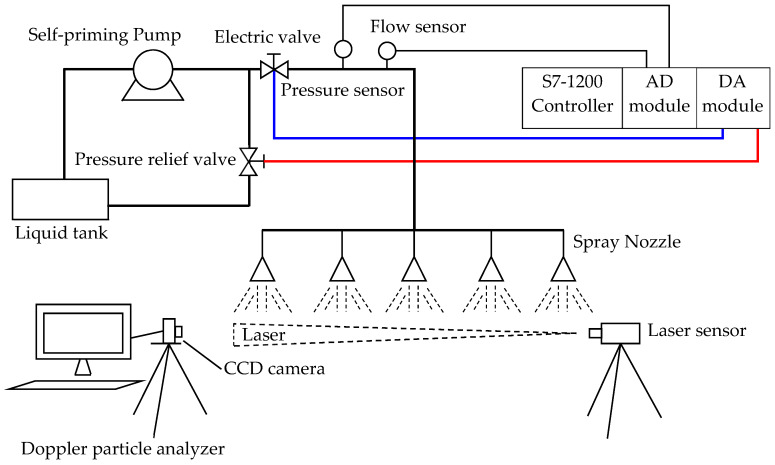
A schematic diagram of the spray test bench.

**Figure 9:**
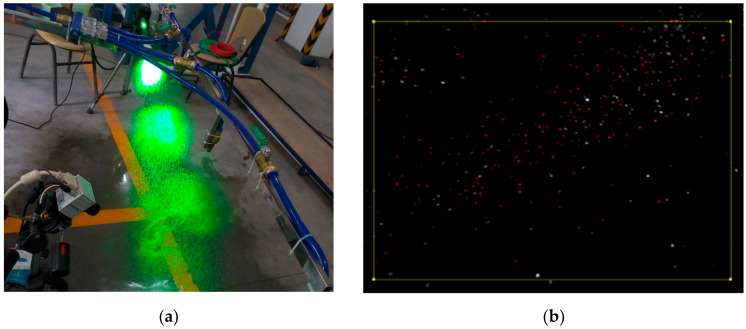
Doppler particle sensor experiment. (**a**) Doppler particle sensor measuring droplet size; (**b**) Doppler particle sensor capturing particle image.

In the actual agricultural device for variable spray purposes, a spray test bench was established based on the performance index. The basic structure of the spray test bench is shown in Figure 8. The pressure sensor (5) and the flow sensor (6) fed back the pressure and flow of the system to the PLC control system. The flow sensor signal was combined with the dose required by the crop as the input of the chaotic optimization composite controller. The output of the chaotic optimization composite controller and the input of the pressure sensor were simultaneously used as the input in the control over the variable spray valve. The output of the variable spray valve passed through the pressure sensor, the flow sensor, and the nozzle in order to act on crops. Both the red pressure circuit and the blue flow circuit were controlled to achieve the so-called double closed loop control. Particle diameter analysis was performed on the droplet particles in the fog field using a Doppler dynamic particle image velocimeter. PIV (Doppler particle image velocimeter) consists of an illumination laser, a synchronous controller, an image acquisition board (placed in a computer), a high-speed digital camera, and a computer. The laser emitted a laser beam through a composite lens to expand into a piece of light illumination flow field, which facilitated the shooting of particles. Figure 9a is a graph showing the velocity and magnitude of a Doppler dynamic PIV using a laser to measure variable spray droplets. Figure 9b shows the dynamic image of the variable spray particles captured by the PIV. By comparison of the calibration, pixel grayscale threshold setting, and pixel point comparison, the size and speed of the variable spray droplets were obtained.

By writing a program in Siemens S7-1200PLC, the Bang-Bang control program and the optimized fuzzy PID program were implanted into the PLC. The PID controller was responsible for outputting an accurate value signal to the motor depending on the three parameters (Δ$K_{p}$, Δ$K_{i}$, Δ$K_{d}$) of the real-time setting output. The actual flow rate of the flow valve was controlled by the motor armature voltage.

### 4.2. Experimental Results

In the actual agricultural spray application, expert experience tells us that the spray application effect is often best when the droplet diameter is in the range of 100–300 µm. Therefore, in this experiment, the particle diameter was 1–4 pixels, that is, the actual diameter was 78–312 µm. The goal of the measuring operation was to test the size of the spray droplet particles diameter at different pressures and flows. According to the system configuration, the pressure was set at 0.5 Mpa, 0.4 Mpa, 0.3 Mpa, 0.2 Mpa, and 0.1 Mpa, and the flow rate was 0.1 m^3^/h, 0.08 m^3^/h, 0.07 m^3^/h, 0.06 m^3^/h, 0.05 m^3^/h, and 0.04 m^3^/h. Under such measurement conditions, of course, the actual value of the pressure and flow adjustment fluctuated above and below the set value, and then the average value was obtained. Since the error of the arithmetic mean was 0.5 times smaller than the error of a single observation, the arithmetic mean was more reliable. Each data point was measured three times at the same pressure and flow rate, and averaged. Under the pressure at 0.5 Mpa, the maximum flow rate of the variable spray control system was as high as approximately 0.10m^3^/h. As the figure rose to around 0.07 m^3^/h, the effective particle number of the variable spray control system was found significantly reduced. This made the pressure at 0.5 Mpa and the flow rate in the range of 0.10 m^3^/h to 0.07 m^3^/h the effective measurement interval of particles. With a flow rate of 0.10 m^3^/h, the effective number of particles in the optimized fuzzy adaptive PID control system, i.g., the number of particles between 1 and 4 was 427 (The effective particle number of the PID control system set as 337), and the total particle number is 522 (the effective particle number of the PID control system is 430) as shown in Table 4. The ratio of the number of effective particles to the total number of particles was therefore registered as 81.8% as shown in Table 4. For the flow rate of 0.07 m^3^/h, this ratio at its maximum level would get 84.5% (the highest PID control system ratio set as 83.6%), where the total number of particles and the number of effective particles were down to the lowest level, as shown in Table 4.

Under the pressure at 0.4 Mpa, the effective range of the flow rate of the variable spray control system may be the same as the pressure at 0.5 Mpa. As the flow rate came up to 0.07 m^3^/h, the maximum number of effective particles in the total number of particles was registered as 83.8% (PID control system ratio set as 73.5%), as shown in Table 5. Note that in such scenario, the total number of effective particles to the pressure was merely 34, as shown in Table 5. By the time when the flow rate was stepped up to 0.10, the second highest proportion of effective particles to the total number of particles was recorded as 82.5% (the proportion of PID control system set as 80.8%), as shown in Table 5. Correspondingly, the number of effective particles was 231 in total (the total number of effective particles of the PID control system set as 181), as shown in Table 5.

Under the pressure of 0.3 Mpa, the maximum flow rate of the variable spray control system was approximately 0.09 m^3^/h. When the flow was around 0.06 m^3^/h, the variable spray control system got the number of effective particles being significantly reduced. Under this pressure, the flow rate from 0.09 m^3^/h to0.06 m^3^/h provided a range available for the particle effective measurement interval. For the flow rate of 0.06 m^3^/h, the maximum number of effective particles to the total number of particles went as high as 89.4% (the proportion of PID control system as 81.3%), as shown in Table 6. In this scenario, the total number of effective particles went up to 397 (the total number of effective particles of the PID control system set as 309), as shown in Table 6. As the flow rate was marked 0.09 and 0.07, respectively, the second highest ratio of effective particles to the total number of particles was the same as 83.6% (the PID control system ratio was 82.0% and 83.0%), as shown in Table 6. The corresponding total effective particles were 464 and 219 (the total effective particles of the PID control system set as 359 and 171), as shown in Table 6.

Under the pressure of 0.2 Mpa, the maximum flow rate of the variable spray control system was approximately 0.07 m^3^/h. When the flow was around 0.04 m^3^/h, the variable spray control system got the number of effective particles being significantly reduced. Under this pressure, the flow rate from 0.07 m^3^/h to0.04 m^3^/h provided a range available for the particle effective measurement interval. When the flow rate was 0.06 m^3^/h, the proportion of the effective particles to the total particles was 86.4% (the proportion of PID control system as 82.6%), as shown in Table 7. but the corresponding total effective particles under the flow rate and pressure was only 140 (the total effective particles of PID control system as 109), as shown in Table 7.

Under the pressure of 0.1 Mpa, the maximum flow rate of the variable spray control system was about 0.05 m^3^/h. When the flow rate was about 0.05 m^3^/h, the effective particle number of the variable spraying control system was obviously reduced. When the flow rate was 0.05 m^3^/h, the ratio of effective particle number to total particle number was 85% (the ratio of PID control system as 83.1%), as shown in Table 8, but the total number of effective particles corresponding to the flow rate and pressure was the same. Only 153 (the total number of effective particles in PID control system as 118), as shown in Table 8. Therefore, when the pressure was less than 0.1 MPa, the effective particle number of the system couldn’t meet the requirements, so the experiment was over.

**Figure 10:**
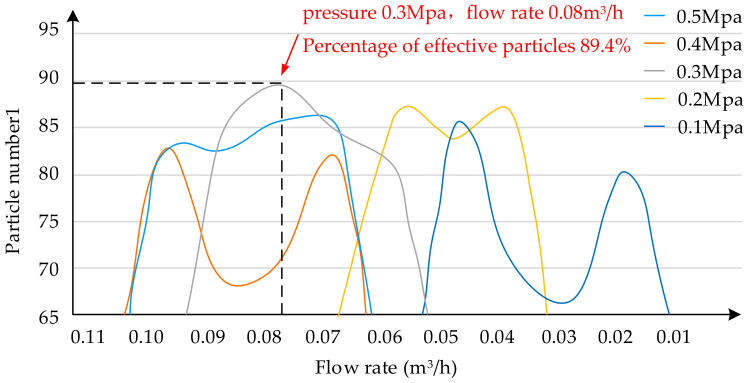
Percentage of spray droplets taking up the total droplets at each flow rate at 0.5 Mpa, 0.4 Mpa, 0.3 Mpa, 0.2 Mpa and 0.1 Mpa.

According to the experimental data, calculate the proportion of effective droplets to total droplets at different flows under the same pressure, and draw the distribution curve of the effective droplets. The *X*-axis represents the spray flow, and the *Y*-axis represents the percentage of the total droplets at the effective droplet station, as shown in Figure 10, where the pressures corresponding to the five curves are 0.5 Mpa, 0.4 Mpa, 0.3 Mpa, 0.2 Mpa, 0.1 Mpa, respectively. Each curve indicates the percentage relationship between the spray flow rate and the total droplets of an effective droplet station at the same pressure. As clearly shown in Figure 10, the pressure at 0.3 Mpa and the flow rate at 0.08 m^3^/h are combined to take out the effective droplet percentage up to 89.4%, ensuring that the spray effect gets the optimal level.

**Figure 11:**
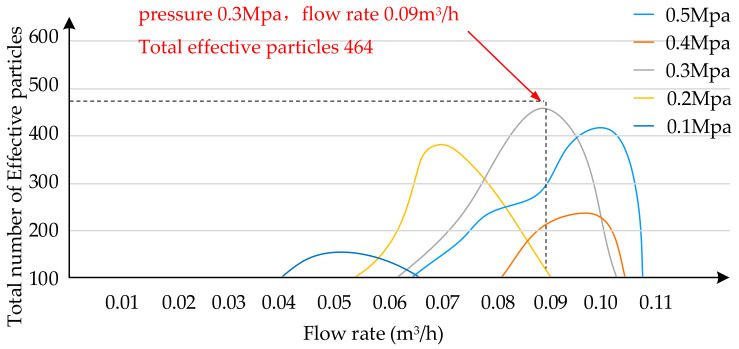
Total effective droplets at each flow rate at 0.5 Mpa, 0.4 Mpa, 0.3 Mpa, 0.2 Mpa and 0.1 Mpa.

According to the experimental data, record the relationship of the total effective droplets at different flows under the same pressure, and draw the distribution curve of the total effective droplets. The *X*-axis is the spray flow and the *Y*-axis the total amount of effective mist droplets as plotted in Figure 11. The five curves herein respectively show the relationship between the spray flow rate and the effective total amount of droplets at the same pressure. Apparently, the combination of the pressure at 0.3 Mpa and the flow rate at 0.09 m^3^/h is able to generate the total effective droplet volume to the highest level, exhibiting the spray volume at its optimal status.

## 5. Conclusions

In this work, a compound controller and a double closed-loop control system were designed for the variable spray control system. To get better control parameters, the chaos algorithm of the compound controller was optimized accordingly. The double closed loop control system worked with pressure as the inner loop and flow as the outer loop. The variable control spray system was then introduced, and supported by simulation for PID control with the help of chaos optimization compound control. The simulation results showed that after chaotic optimized composite control, the system’s dynamic performance displayed superior indices to those under the conventional PID control. To further verify the effectiveness of the control system, a variable spray control system was established, and the controller parameters optimized by chaos were programmed into the PLC controller. The control system was placed through a Doppler dynamic particle analyzer. First, under a constant pressure, as the flow rate of the variable spray system decreased, the atomization effect of the spray became worse, and the spray droplets captured by the Doppler dynamic particle sensor presented a downward trend. Secondly, for too small a pressure as it was, the spray effect of the variable spray system got even worse. At different flow rates, the effect of improving the spray effect was not so obvious. Third, the higher the pressure, the better the spray effect. Note that if the pressure got too high, the particle size of the variable spray system would increase significantly and the percentage of effective droplets decreased. Fourth, given the pressure of 0.4 Mpa, 0.3 Mpa, 0.2 Mpa and 0.1 Mpa, respectively, the decrease of the flow rate may lead to an increase in the proportion of spray droplets with unqualified particle sizes. Since the image magnification of the PIV device is 78 μm/pixel, the droplets to be captured during the measurement were between 1 and 5, which may cause a large error. As a result, digital camera may become worse and worse by capturing images with greater randomness. Therefore, the above data is for research reference only. Fifth, the proposed control system significantly increased the number of effective particles in the variable spray system, wherein the number of effective particles with a diameter of 100–300 μm rose to 89.4%. This has greatly exceeded the effect that the conventional PID control may have and can be used as a powerful control method for precise control of agricultural variable spray.

## Figures and Tables

**Table 1:** Fuzzy control rules for Δ$K_{p}$.

$\Delta K_{p}$		${\dot{e}}_{Q}\left(t\right)$						
		NB	NM	NS	ZO	PS	PM	PB
$e_{Q}\left(t\right)$	NB	PB	PB	PM	PM	PS	ZO	ZO
	NM	PB	PB	PM	PS	PS	ZO	NS
	NS	PM	PM	PM	PS	ZO	NS	NS
	ZO	PM	PM	PS	ZO	NS	NM	NM
	PS	PS	PS	ZO	NS	NS	NM	NM
	PM	PS	ZO	NS	NM	NM	NM	NB
	PB	ZO	ZO	NM	NM	NM	NB	NB

**Table 2:** Fuzzy control rules for Δ$K_{i}$.

$\Delta K_{i}$		${\dot{e}}_{Q}\left(t\right)$						
		NB	NM	NS	ZO	PS	PM	PB
$e_{Q}\left(t\right)$	NB	NB	NB	NM	NM	NS	ZO	ZO
	NM	NB	NB	NM	NS	NS	ZO	ZO
	NS	NB	NM	NS	NS	ZO	PS	PS
	ZO	NM	NM	NS	ZO	PS	PM	PM
	PS	NM	NS	ZO	PS	PS	PM	PB
	PM	ZO	ZO	PS	PS	PM	PB	PB
	PB	ZO	ZO	PS	PM	PM	PB	PB

**Table 3:** Fuzzy control rules for Δ$K_{d}$.

$\Delta K_{d}$		${\dot{e}}_{Q}\left(t\right)$						
		NB	NM	NS	ZO	PS	PM	PB
$e_{Q}\left(t\right)$	NB	NM	NB	NB	NB	NB	NM	NS
	NM	NS	NM	NB	NM	NM	NS	NS
	NS	NS	NS	NM	NM	NS	NS	ZO
	ZO	ZO	NS	NS	NS	NS	NS	ZO
	PS	ZO	ZO	ZO	ZO	ZO	ZO	ZO
	PM	PB	PS	PS	PS	PS	PS	PB
	PB	PB	PM	PM	PM	PS	PS	PB

**Table 4:** Spray conditions at various flow rates at the pressure of app. 0.5 MPa.

Number of Particles	Flow Rate m^3^/h							
	0.10 m^3^/h		0.09 m^3^/h		0.08 m^3^/h		0.07 m^3^/h	
	CFC	PID	CFC	PID	CFC	PID	CFC	PID
1~2	182	145	142	101	101	81	84	65
2~3	133	104	68	86	88	72	83	64
3~4	112	88	79	50	49	38	45	34
4~5	64	60	45	48	36	35	28	21
5 or more	26	33	17	26	18	18	11	11
1~4 (Effective particles)	427	337	289	237	238	191	212	163
Total number of particles	522	430	351	311	292	244	251	195
Effective particles percentage	81.8%	78.4%	82.3%	76.2%	81.5%	78.3%	84.5%	83.6%

Note: CFC is chaos optimization for adaptive fuzzy controller and variable domain fuzzy controller.

**Table 5:** Spray conditions at various flow rates at the pressure of app. 0.4 MPa.

Number of Particles	Flow Rate m^3^/h							
	0.10 m^3^/h		0.09 m^3^/h		0.08 m^3^/h		0.07 m^3^/h	
	CFC	PID	CFC	PID	CFC	PID	CFC	PID
1~2	76	60	96	75	41	31	15	12
2~3	83	65	57	44	25	20	14	12
3~4	72	56	49	40	9	7	2	1
4~5	36	30	37	31	14	10	3	3
5 or more	13	13	54	48	17	16	3	6
1~4 (Effective particles)	231	181	202	159	75	58	31	25
Total number of particles	280	224	293	238	106	84	37	34
Effective particles percentage	82.5%	80.8%	68.9%	66.8%	70.8%	69.0%	83.8%	73.5%

**Table 6:** Spray conditions at various flow rates at the pressure of app. 0.3 MPa.

Number of Particles	Flow Rate m^3^/h							
	0.09 m^3^/h		0.08 m^3^/h		0.07 m^3^/h		0.06 m^3^/h	
	CFC	PID	CFC	PID	CFC	PID	CFC	PID
1~2	207	164	185	144	100	79	63	49
2~3	149	112	138	110	63	48	38	29
3~4	108	83	74	55	56	44	36	26
4~5	56	48	31	41	35	30	22	20
5 or more	36	31	16	30	6	5	12	11
1~4 (Effective particles)	464	359	397	309	219	171	137	104
Total number of particles	555	438	444	380	262	206	170	135
Effective particles percentage	83.6%	82.0%	89.4%	81.3%	83.6%	83.0%	80.6%	77.0%

**Table 7:** Spray conditions at various flow rates at the pressure of app. 0.2 MPa.

Number of Particles	Flow Rate m^3^/h							
	0.07 m^3^/h		0.06 m^3^/h		0.05m^3^/h		0.04 m^3^/h	
	CFC	PID	CFC	PID	CFC	PID	CFC	PID
1~2	206	164	68	55	40	32	20	16
2~3	80	60	40	32	17	12	11	9
3~4	104	82	32	22	10	8	0	2
4~5	111	90	15	16	6	5	2	3
5 or more	100	90	7	7	7	7	3	2
1~4 (Effective particles)	390	306	140	109	67	52	31	27
Total number of particles	600	486	162	132	79	64	36	32
Effective particles percentage	65.0%	63.0%	86.4%	82.6%	84.8%	81.3%	86.1%	84.4%

**Table 8:** Spray conditions at various flow rates at the pressure of app. 0.1 MPa.

Number of Particles	Flow Rate m^3^/h							
	0.05 m^3^/h		0.04 m^3^/h		0.03 m^3^/h		0.02 m^3^/h	
	CFC	PID	CFC	PID	CFC	PID	CFC	PID
1~2	67	53	21	16	15	12	10	8
2~3	51	39	9	6	7	5	6	4
3~4	35	26	5	4	4	3	0	0
4~5	14	12	6	5	6	5	1	1
5 or more	13	12	9	8	7	6	3	2
1~4 (Effective particles)	153	118	35	26	26	20	16	12
Total number of particles	180	142	52	39	40	31	20	15
Effective particles percentage	85.0%	83.1%	67.3%	66.7%	65.0%	64.5%	80.0%	80.0%
